# Molecular detection of omicron SARS-CoV-2 variant is achieved by RT-LAMP despite genomic mutations

**DOI:** 10.1590/0074-02760220050

**Published:** 2022-06-22

**Authors:** Letícia Trindade Almeida, Amanda Bonoto Gonçalves, Ana Paula Moreira Franco-Luiz, Thais Bárbara de Souza Silva, Pedro Augusto Alves, Rubens Lima do Monte-Neto

**Affiliations:** 1 Fundação Oswaldo Cruz-Fiocruz, Instituto René Rachou, Biotecnologia Aplicada a Patógenos, Belo Horizonte, MG, Brasil; 2 Fundação Oswaldo Cruz-Fiocruz, Instituto René Rachou, Imunologia de Doenças Virais, Belo Horizonte, MG, Brasil

**Keywords:** RT-LAMP, omicron, SARS-CoV-2, mutations, *N* and *E* genes

## Abstract

**BACKGROUND:**

Severe acute respiratory syndrome coronavirus (SARS-CoV-2) omicron variant was first detected in South Africa in November 2021. Since then, the number of cases due to this variant increases enormously every day in different parts of the world. Mutations within omicron genome may impair the molecular detection resulting in false negative results during Coronavirus disease 19 (COVID-19) diagnosis.

**OBJECTIVES:**

To verify if colorimetric reverse transcription loop-mediated isothermal amplification (RT-LAMP) targeting *N* and *E* genes would work efficiently to detect omicron SARS-CoV-2 variant and its sub-lineages.

**METHODS:**

SARS-CoV-2 reverse transcription quantitative polymerase chain reaction (RT-qPCR) positive samples were sequenced by next generation DNA sequencing. The consensus sequences generated were submitted to Pangolin tool for SARS-CoV-2 lineage identification. RT-LAMP reactions were performed at 65ºC/30 min targeting *N* and *E.*

**FINDINGS:**

SARS-CoV-2 omicron can be detected by RT-LAMP targeting *N* and *E* genes despite the genomic mutation of this more transmissible lineage. Omicron SARS-CoV-2 sub-lineages were tested and efficiently detected by RT-LAMP. We demonstrated that this test is very sensitive in detecting omicron variant, with LoD as low as 0.4 copies/µL.

**MAIN CONCLUSIONS:**

Molecular detection of omicron SARS-CoV-2 variant and its sub-lineages can be achieved by RT-LAMP despite the genomic mutations as a very sensitive surveillance tool for COVID-19 molecular diagnosis.

On November 24, 2021 the severe acute respiratory syndrome coronavirus (SARS-CoV-2), PANGO lineage B.1.1.529, was first reported in Botswana and South Africa, pummeling the world by the beginning of December 2021. The World Health Organization (WHO) named B.1.1.529 as omicron and classified as the fifth variant of concern (VOC).^1^ Omicron has mutations in genomic coding regions to structural (envelope, nucleocapsid, membrane, spike) and nonstructural proteins that can impair Coronavirus disease (COVID-19) molecular diagnosis based on reverse transcription quantitative polymerase chain reaction (RT-qPCR) tests, leading to misdiagnosis and allowing virus spreading through false negative results.^1^ ^,^ ^2^ ^,^ ^3^ As an alternative to RT-qPCR, reverse transcription loop-mediated isothermal amplification (RT-LAMP) has been used with success to detect SARS-CoV-2 RNA from nasopharyngeal swabs and saliva samples.^4^ During RT-LAMP reaction, the amplification of genetic material occurs at constant temperature (65ºC) without the need for sophisticated thermal cyclers as in RT-qPCR.^5^ Furthermore, the colorimetric reaction allows the result to be interpreted faster than RT-qPCR, since the amplified products can be visually detected due to pH-dependent sensors that changes reaction color from pink to yellow (positive result).^6^ Previous results obtained by our group, showed that RT-LAMP reaction targeting SARS-CoV-2 *N* and *E* genes, can be equivalent to the gold standard RT-qPCR using nasopharyngeal swabs samples.^4^ Given the fact that genomic mutations in SARS-CoV-2 omicron VOC are also present in *N* and *E* coding sequences, the main goal of this work was to verify whether RT-LAMP reaction targeting *N* and *E* genes (in a multiplex or singleplex manner), would works to detect the virus reinforcing the use of it as an affordable and robust COVID-19 diagnostic tool.

## MATERIALS AND METHODS

Clinical samples obtained from nasopharyngeal swabs were collected in different health units in the municipality of Belo Horizonte and its surrounding metropolitan region, Brazil. Molecular diagnosis using RT-qPCR (Allplex™ SARS-CoV-2 - Seegene, South Korea) - which targets the *E*, *S*/*RdRp* and *N* genes of SARS-CoV-2 - was performed in the municipal molecular biology laboratory of Belo Horizonte city hall. Twenty positive samples were selected for DNA sequencing and RT-LAMP analysis. For next generation DNA sequencing, the libraries were constructed using Illumina COVIDSeq™ Test (Illumina Inc, San Diego, CA, USA), according to manufacturer’s instructions, and the samples were sequenced through Illumina MiSeq Platform. The trimming tool used in the raw reads was the Trimmomatic^7^ with a sliding window of four nucleotides with an average Phred score of 20, and filtered reads smaller than 50 bp were removed. The trimmed reads were aligned to the SARS-CoV-2 reference genome (NC_045512) using BWA^8^ with the default parameters. The nucleotide variants were detected using iVar package,^9^ with a minimum frequency of 50% and depth of 30 reads. To identify the SARS-CoV-2 lineage and sub-lineages, the consensus sequences generated were submitted to Pangolin tool v3.1.17 (http://pangolin.cog-uk.io/).^10^ All sample sequences were deposited at the European Nucleotide Archive, under project number PRJEB49204, and at GISAID Initiative [Supplementary data (Table)]. The RT-LAMP test was performed in a singleplex way with the set of primers targeting independently *N* (primer set *N2*) and *E* (primer set *E1*) genes or multiplexed (combined primer sets *N2*/*E1*). Detailed conditions of RT-LAMP reaction, including primer sequences, are described in our previous work.^4^ Briefly, reactions were performed at 65ºC, during 30 min, using WarmStart^®^ Colorimetric LAMP 2× Master Mix (New England Biolabs #M1804). To validate the visual colorimetric output, samples resolved in 2% agarose gel stained with DNA intercalator GelRed^®^ (Biotium #41003), confirming the specific amplification when compared to the positive control (RNA of inactivated parental lineage B of SARS-CoV-2 extracted from the supernatant infected Vero E6 cells). The test of the limit of detection (LoD) was performed by RT-LAMP using as input a serial dilution of one of the omicron samples, and the absolute quantification was conducted based on a standard curve prepared using the template SARS-CoV-2 *E* gene-harboring plasmid (2 × 10^5^ copies/µL; Biogene COVID-19 PCR, Bioclin/Quibasa #K228-1; Lot: 0007). More details of the curve and how the LoD was performed are described in our previous work.^4^ Sequencing alignments were performed on SnapGene using embedded multiple alignment Clustal Omega tool. NCBI NC_045512 was included as SARS-CoV-2 reference genomic sequence.

*Ethics statement* - The studies involving human participants were reviewed and approved by Research Ethics Committee involving human beings at Instituto René Rachou, Fundação Oswaldo Cruz, under license protocol number: 4084902 and CAAE (certificate of presentation for ethical appreciation): 31984720300005091.

## RESULTS

**Fig. 1:**
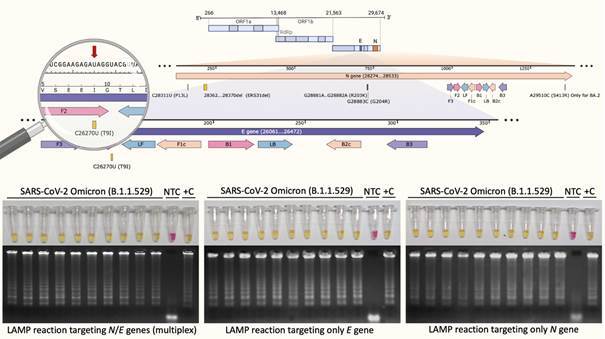
colorimetric reverse transcription loop-mediated isothermal amplification (RT-LAMP) targeting *N* and *E* genes allows the detection of severe acute respiratory syndrome coronavirus (SARS-CoV-2) variant omicron. Selected nasopharyngeal swab-derived samples, previously detected positive for SARS-CoV-2 by reverse transcription quantitative polymerase chain reaction (RT-qPCR) and identified as the new omicron variant of concern (VOC) by next generation sequencing, were included as input for colorimetric RT-LAMP reaction targeting *N* and *E* genes. RT-LAMP reactions were performed combining the primer sets *N*2/*E*1 in the same reaction (multiplex) or independently.^(4)^ Both strategies resulted in positive reaction as evidenced by yellow colour. Negative or absence of specific amplification is represented by pink colour and no bands on gel electrophoresis. RT-LAMP reaction was performed at 65ºC during 30 min, using the WarmStart^®^ colorimetric LAMP 2X master mix (NEB #M1804). RT-LAMP amplification products were resolved in 2% agarose gel and stained with GelRed^®^ (Biotium #41003) to confirm DNA amplification. +C: positive control using SARS-CoV-2 RNA extracted from laboratory-cultured inactivated SARS-CoV-2; NTC: non-template control. Upper panel cartoon is showing SARS-CoV-2 genome where *N* and *E* gene regions are zoomed in. Polymorphisms are indicated in their corresponding positions. The SNP C26270U, highlighted by the red arrow, is the only polymorphism that overlaps with F2 primer (magnifying glass). F3: forward; B3: backward; LF: loop forward; LB: loop backward; F2 and F1c: parts of FIP - forward internal primer; B1 and B2c: parts of BIP - backward inner primer. P: proline; L: leucine; R: arginine; K: lysine; G: glycine; S: serine; T: threonine; I: isoleucine; E: glutamic acid. BA.2, also known as 21L, is considered a sub-lineage of SARS-CoV-2 omicron. Genomic representation was created using SnapGene. Parts of this figure were created with BioRender.com and are licensed under the agreement number: QC23MCKTFE.

**Fig. 2:**
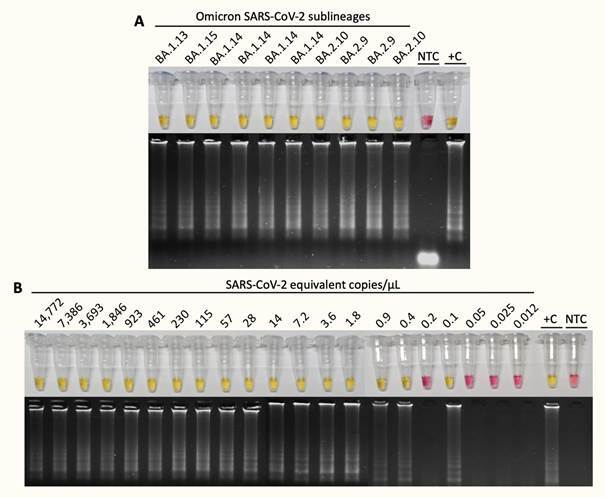
reverse transcription loop-mediated isothermal amplification (RT-LAMP) of Omicron variant sub-lineages. (A) The test was performed using multiplex *N*/*E* strategy. All samples were positive (yellow colour) after 30 min reaction. RT-LAMP amplification products were resolved in 2% agarose gel and stained with GelRed^®^ (Biotium #41003) to confirm DNA amplification. +C: positive control using severe acute respiratory syndrome coronavirus (SARS-CoV-2) RNA extracted from laboratory-cultured inactivated SARS-CoV-2; NTC: non-template control. From the left to the right hand side, clinical samples corresponds to Supplementary data (Table samples 11 to 20. (B) Limit of detection (LoD) of Omicron samples. The test was performed using as input a serial dilution of the sample 3 (randomly chosen). The absolute quantification was performed based on a standard curve prepared using the template SARS-CoV-2 *E* gene-harboring plasmid 2 × 10^5^ copies/µL; Biogene Coronavirus disease 19 (COVID-19) polymerase chain reaction (PCR), Bioclin/Quibasa #K228-1; Lot: 0007. The equation of a straight line is y = -3,476x+38,063, where Y is the Ct for *E* gene of the sample 3 in reverse transcription quantitative polymerase chain reaction (RT-qPCR) and X is the value of quantification. RT-LAMP amplification products were resolved in 2% agarose gel and stained with GelRed^®^ (Biotium #41003) to confirm DNA amplification. +C: positive control using SARS-CoV-2 RNA extracted from laboratory-cultured inactivated SARS-CoV-2; NTC: non-template control. Figure representative of independent assays and signs of cutted and overlapping images can be seen for this reason. Original raw material can be send upon request.

Aiming to give experimental evidence that RT-LAMP tests using multiplex strategy (*N*2/*E*1) or *N*2 and *E*1 primer sets alone are able to detect B.1.1.529 SARS-CoV-2 lineage, we performed the RT-LAMP tests using RT-qPCR positive samples that were confirmed as omicron VOC by whole genome sequencing. Through sequencing, these samples were classified in Omicron sub-lineages (ten BA.1.14, four BA.1.15, two BA.2.10, two BA.2.9, one BA.1.13 and one BA.1.18). The ten BA.1.14 were positive in RT-LAMP when the target was multiplex *N*/*E* or the *E* e *N* separately (singleplex) turning the original pink color into yellow, as the colorimetric evidence for specific DNA amplification (Fig. 1). Since there was no difference between multiplex or singleplex strategy we performed the multiplex *N*/*E* RT-LAMP for the other sub-lineages samples. As demonstrated in Fig. 2, all tubes became yellow after the reaction. The LoD test demonstrated that RT-LAMP using the *N*/*E* multiplex is efficient for detection of Omicron samples containing as low as 0.4 copies/µL (Fig. 2B). In summary, either RT-LAMP strategies (multiplex or not) worked to successfully detect omicron RNA. The omicron-derived genomic sequences were aligned against the reference SARS-CoV-2 genome together with RT-LAMP primer sets targeting *N* and *E* genes. None of the mutations present in omicron affects *N2* primer recognition, since the target region do not overlaps with any of the six nonsynonymous mutations in *N* gene (Fig. 1). By contrast, the only mutation present in *E* gene region - a single nucleotide polymorphism (SNP) resulting in a C-to-U transition at position 26270 (C26270U), that yields amino acid change T9I - overlaps with *E1* forward inner primer F2 part, apparently without any impact on primer recognition and specific amplification by RT-LAMP (Fig. 1).

## DISCUSSION

The circulation of new SARS-CoV-2 variants is challenging for diagnostic tests since these strains accumulate mutations that can affect primer or probe recognition, leading to false negative results in standard RT-qPCR diagnostic tests. Indeed, molecular diagnostic test sensitivity is intrinsically related to the target choice. Mismatches could affect reaction’s efficiency, especially if it occurs at the 3’-end of a primer.^11^ Studies demonstrated that RT-qPCR can fail in detecting omicron VOC, yielding false negative results.^2^ ^,^ ^12^ ^,^ ^13^ Lesbon et al.^2^ reported that high viral load samples (Ct < 33) could be detected when targeting *E* and *RdRp* genes but failed when considering *N* gene as target, even the latter being considered the most sensitive region for RT-qPCR.^2^ In addition, the mutation C26340U at *E* gene was associated with a failure on detecting SARS-CoV-2 by RT-qPCR,^13^ compromising RT-qPCR-based SARS-CoV-2 detection kits such as GeneFinder^TM^ COVID-19 Plus RealAmp Kit and cobas^®^ SARS-CoV-2 test.^2^ ^,^ ^12^ ^,^ ^13^ These evidences lead us to wonder if omicron genomic polymorphisms would affect molecular detection by RT-LAMP. Until now, there is no evidence that mutations in SARS-CoV-2 *N* or *E* genes would affect omicron detection by RT-LAMP. Indeed, here we report that the only detected potential mismatch in *E* gene (mutation C26270U) does not impair specific recognition by *E1* primer set, resulting in successful target amplification. Concerning mutations in *N* gene, Bei et al.^14^ reported that the presence of C28311U SNP, that overlaps N1 probe, does not impact omicron detection by CDC 2019-nCoV_N1 primer-probe set from the US Centre for Disease Control and Prevention (CDC) RT-qPCR-based SARS-CoV-2 detection kit.^14^ None of the mutations present in *N* gene perturb RT-LAMP based omicron detection, since *N2* primer set is based in a conserved region. It is worth to note that SNPs C28311U, G28881A, G28882A, G28883C in *N* gene are ancestral and present in alpha (B1.1.7) and Gamma (P.1) SARS-CoV-2 VOCs.^15^ ^,^ ^16^ The latter are derive from a trinucleotide substitution GGG > AAC yielding nucleocapsid mutation R203K and G204R, linked to increased: (I) RNA expression^17^ and (II) viral load^18^ (https://covariants.org/variants/21K.Omicron and https://covariants.org/variants/21L.Omicron).

The LoD test demonstrated that RT-LAMP using the *N*/*E* multiplex detected at least 0.4 copies/µL of a Omicron sample. Moreover, the RT-LAMP was efficient when six different sub-lineages of Omicron variant were used as input. In this regard, RT-LAMP works as a highly sensitive and specific molecular test to detect SARS-CoV-2 variants.^4^ This work highlights the importance of RT-qPCR constant monitoring to detect current circulating SARS-CoV-2 and reinforces RT-LAMP as a robust alternative for massive COVID-19 surveillance.
