# Supplementary material for: Molecular detection of omicron SARS-CoV-2 variant is achieved by RT-LAMP despite genomic mutations
Source: Mem Inst Oswaldo Cruz. 2022 Jun 22;117:e220050. doi: 10.1590/0074-02760220050 (PMC9219828; doi:10.1590/0074-02760220050)
Supplement: Supplementary file 1 [file 1678-8060-mioc-117-e220050-s.pdf]

TABLE

Data of the samples used in the study. The samples were sequenced by Next Generation Sequencing (NGS), on the Illumina MiSeq platform. All sequences were deposited in the GISAID database. Pangolin (Pango v.4.0.4 PLEARN-v1.3) was used to identify the variants. AA: aminoacid; NP: Not Performed.

Sample	Ct E gene	Ct E RdRp	Ct N gene	Collection date	Collection location	Gender	Patient age	Accession ID GISAID	Lineage	Sublineage	AA Substitutions	Coverage
1	24.55	28.15	24.04	2021-12-27	South America / Brazil / Minas Gerais / Esmeraldas	Male	39	EPI_ISL_9274974	VOC Omicron GRA (B.1.1.529+BA.)	BA.1.15	Spike D614G, Spike D796Y, Spike G142D, Spike G339D, Spike H655Y, Spike L981F, Spike N679K, Spike N764K, Spike N856K, Spike N969K, Spike P681H, Spike Q954H, Spike T95I, Spike T478K, Spike T547K, Spike V143del, Spike Y144del, Spike Y145del, E T9I, M A63T, M D3G, N G204R, N P13L, N R203K, NS7b T40I, NSP3 A1892T, NSP3 K38R, NSP3 L1266I, NSP3 S1265del, NSP4 T492I, NSP5 P132H, NSP6 I189V, NSP12 P323L, NSP14 I42V	651.862x
2	24.36	28.40	24.75	2021-12-23	South America / Brazil / Minas Gerais / Belo Horizonte	Female	57	EPI_ISL_9340194	VOC Omicron GRA (B.1.1.529+BA.)	BA.1.18	Spike D614G, Spike H655Y, Spike L452R, Spike L981F, Spike N679K, Spike N969K, Spike P681H, Spike Q954H, Spike T95I, Spike T478K, E T9I, M A63T, M D3G, N G204R, N P13L, N R203K, NS7a V82A, NS7b T40I, NSP3 A1892T, NSP3 K38R, NSP4 T492I, NSP12 P323L	756.638x
3	23.57	26.62	23.40	2021-12-23	South America / Brazil / Minas Gerais / Belo Horizonte	Male	62	EPI_ISL_9274978	VOC Omicron GRA (B.1.1.529+BA.)	BA.1.15	Spike D215E, Spike D614G, Spike D796Y, Spike G142D, Spike G339D, Spike H655Y, Spike ins215PEX, Spike L981F, Spike N679K, Spike N764K, Spike N856K, Spike N969K, Spike P681H, Spike Q954H, Spike T95I, Spike T547K, Spike V143del, Spike Y144del, Spike Y145del, E T9I, M A63T, M D3G, M Q19E, N G204R, N P13L, N R203K, NS7b T40I, NSP2 T149I, NSP3 A1892T, NSP3 K38R, NSP3 L1266I, NSP3 S1265del, NSP4 T492I, NSP5 P132H, NSP6 I189V, NSP12 P323L, NSP14 I42V	1137.59x
4	20.87	24.46	20.86	2021-12-27	South America / Brazil / Minas Gerais / Belo Horizonte	Male	46	EPI_ISL_9274981	VOC Omicron GRA (B.1.1.529+BA.)	BA.1.14	Spike D614G, Spike D796Y, Spike G142D, Spike G339D, Spike H655Y, Spike L981F, Spike N501Y, Spike N679K, Spike N764K, Spike N856K, Spike N969K, Spike P681H, Spike Q498R, Spike Q954H, Spike T95I, Spike T547K, Spike V143del, Spike Y144del, Spike Y145del, Spike Y505H, E T9I, M A63T, M D3G, N G204R, N P13L, N R203K, NS7b T40I, NSP3 A1892T, NSP3 K38R, NSP3 L1266I, NSP3 S1265del, NSP4 T492I, NSP5 P132H, NSP6 I189V, NSP12 P323L, NSP14 I42V	997.739x
5	26.68	30.06	27.73	2021-12-27	South America / Brazil / Minas Gerais / Belo Horizonte	Male	32	EPI_ISL_9274983	VOC Omicron GRA (B.1.1.529+BA.)	BA.1.14	Spike D614G, Spike D796Y, Spike G142D, Spike G339D, Spike H655Y, Spike L981F, Spike N679K, Spike N764K, Spike N856K, Spike N969K, Spike P681H, Spike Q954H, Spike R158del, Spike T95I, Spike T547K, Spike V143del, Spike Y144del, Spike Y145del, E T9I, M A63T, M D3G, N G204R, N P13L, N R203K, NS7b T40I, NSP2 A336V, NSP3 A1892T, NSP3 K38R, NSP3 L1266I, NSP3 S1265del, NSP4 T492I, NSP5 P132H, NSP6 I189V, NSP9 T34I, NSP12 P323L, NSP14 I42V	576.193x
6	27.09	31.04	27.48	2021-12-28	South America / Brazil / Minas Gerais / Belo Horizonte	Female	60	EPI_ISL_9274985	VOC Omicron GRA (B.1.1.529+BA.)	BA.1.15	Spike D614G, Spike D796Y, Spike G142D, Spike H655Y, Spike L452R, Spike L981F, Spike N679K, Spike N764K, Spike N856K, Spike N969K, Spike P681H, Spike Q954H, Spike T95I, Spike T478K, Spike T547K, Spike V143del, Spike Y144del, Spike Y145del, E T9I, M A63T, M D3G, N G204R, N P13L, N R203K, NS7b T40I, NSP3 A1892T, NSP3 K38R, NSP3 L1266I, NSP3 S1265del, NSP4 T492I, NSP5 P132H, NSP6 I189V, NSP12 P323L, NSP14 I42V	1208.94x
7	20.89	25.02	21.65	2021-12-29	South America / Brazil / Minas Gerais / Belo Horizonte	Female	21	EPI_ISL_9274986	VOC Omicron GRA (B.1.1.529+BA.)	BA.1.14	Spike D215E, Spike D614G, Spike D796Y, Spike G142D, Spike G339D, Spike H655Y, Spike ins215PEX, Spike L981F, Spike N679K, Spike N764K, Spike N856K, Spike N969K, Spike P681H, Spike Q954H, Spike S373P, Spike S375F, Spike T95I, Spike T547K, Spike V143del, Spike Y144del, Spike Y145del, E T9I, M A63T, M D3G, M Q19E, N G204R, N P13L, N R203K, NSP3 A1892T, NSP3 K38R, NSP3 L1266I, NSP3 S1265del, NSP4 T492I, NSP5 P132H, NSP6 I189V, NSP12 P323L, NSP14 I42V	1122.36x
8	27.11	30.83	27.16	2021-12-29	South America / Brazil / Minas Gerais / Belo Horizonte	Female	18	EPI_ISL_9274987	VOC Omicron GRA (B.1.1.529+BA.)	BA.1.14	Spike D614G, Spike D796Y, Spike G142D, Spike G339D, Spike H655Y, Spike L981F, Spike N679K, Spike N764K, Spike N856K, Spike N969K, Spike P681H, Spike Q954H, Spike T95I, Spike T547K, Spike V143del, Spike Y144del, Spike Y145del, E T9I, M A63T, M D3G, M Q19E, N G204R, N P13L, N R203K, NSP2 I251M, NSP3 A1892T, NSP3 K38R, NSP3 L1266I, NSP3 S1265del, NSP4 T492I, NSP5 P132H, NSP6 I189V, NSP12 P323L, NSP14 I42V	633.878x
9	26.75	30.03	26.90	2021-12-30	South America / Brazil / Minas Gerais / Belo Horizonte	Male	49	EPI_ISL_9274988	VOC Omicron GRA (B.1.1.529+BA.)	BA.1.14	Spike D614G, Spike D796Y, Spike G142D, Spike G339D, Spike H655Y, Spike L981F, Spike N679K, Spike N764K, Spike N856K, Spike N969K, Spike P681H, Spike Q954H, Spike T95I, Spike T547K, Spike V143del, Spike Y144del, Spike Y145del, E T9I, M A63T, M D3G, M Q19E, N G204R, N P13L, N R203K, NS7b T40I, NSP2 I251M, NSP3 A1892T, NSP3 K38R, NSP3 L1266I, NSP3 S1265del, NSP4 T492I, NSP5 P132H, NSP6 I189V, NSP12 P323L, NSP14 I42V	755.294x
10	22.07	25.88	22.35	2021-12-29	South America / Brazil / Minas Gerais / Belo Horizonte	Female	60	EPI_ISL_9274989	VOC Omicron GRA (B.1.1.529+BA.)	BA.1.14	Spike D614G, Spike D796Y, Spike G142D, Spike G339D, Spike H655Y, Spike L981F, Spike N679K, Spike N764K, Spike N856K, Spike N969K, Spike P681H, Spike Q954H, Spike T95I, Spike T547K, Spike V143del, Spike Y144del, Spike Y145del, E T9I, M A63T, M D3G, M Q19E, N G204R, N P13L, N R203K, NS3 A110S, NSP2 I251M, NSP3 A1892T, NSP3 K38R, NSP3 L1266I, NSP3 S1265del, NSP4 T492I, NSP5 P132H, NSP6 I189V, NSP12 P323L, NSP14 I42V	419.171x
11	29.375	NP	NP	2021-12-25	South America / Brazil / Minas Gerais / Belo Horizonte	Female	44	EPI_ISL_9747676	VOC Omicron GRA (B.1.1.529+BA.)	BA.1.13	Spike D614G, Spike D796Y, Spike G142D, Spike H655Y, Spike L981F, Spike N679K, Spike N764K, Spike N856K, Spike N969K, Spike P681H, Spike Q954H, Spike T95I, Spike T547K, Spike V143del, Spike Y144del, Spike Y145del, M I82T, N G215C, N P13L, N R203M, NS7a V82A, NS7b T40I, NSP3 A1892T, NSP3 K38R, NSP3 L1266I, NSP3 S1265del, NSP4 T492I, NSP5 P132H, NSP6 I189V, NSP12 P323L, NSP14 I42V	880.138x
12	21.67	25.48	20.40	2022-01-17	South America / Brazil / Minas Gerais / Belo Horizonte	Male	17	EPI_ISL_10706103	VOC Omicron GRA (B.1.1.529+BA.)	BA.1.15	Spike D614G, Spike D796Y, Spike E484A, Spike G142D, Spike G339D, Spike G446S, Spike G496S, Spike H655Y, Spike K417N, Spike L981F, Spike N440K, Spike N501Y, Spike N679K, Spike N764K, Spike N856K, Spike N969K, Spike P681H, Spike Q493R, Spike Q498R, Spike Q954H, Spike S371L, Spike S373P, Spike S375F, Spike S477N, Spike T95I, Spike T478K, Spike T547K, Spike V143del, Spike Y144del, Spike Y145del, Spike Y505H, E T9I, M A63T, M D3G, M Q19E, N G204R, N P13L, N R203K, NSP3 A1892T, NSP3 K38R, NSP3 L1266I, NSP3 S1265del, NSP4 T492I, NSP5 P132H, NSP6 I189V, NSP12 P323L, NSP14 I42V	443.91x
13	22.69	26.63	23.87	2022-01-17	South America / Brazil / Minas Gerais / Belo Horizonte	Female	84	EPI_ISL_10706104	VOC Omicron GRA (B.1.1.529+BA.)	BA.1.14	Spike D614G, Spike D796Y, Spike G142D, Spike G339D, Spike G496S, Spike H655Y, Spike L981F, Spike N679K, Spike N764K, Spike N856K, Spike N969K, Spike P681H, Spike Q493R, Spike Q954H, Spike T95I, Spike T547K, Spike V143del, Spike Y144del, Spike Y145del, E T9I, M A63T, M D3G, M Q19E, N G204R, N P13L, N R203K, NSP2 I251M, NSP3 A1892T, NSP3 K38R, NSP3 L1266I, NSP3 S1265del, NSP4 T492I, NSP5 P132H, NSP6 I189V, NSP12 P323L, NSP14 I42V	330.01x
14	24.92	29.18	25.83	2022-01-17	South America / Brazil / Minas Gerais / Belo Horizonte	Female	60	EPI_ISL_10706105	VOC Omicron GRA (B.1.1.529+BA.)	BA.1.14	Spike D614G, Spike D796Y, Spike G142D, Spike G339D, Spike H655Y, Spike L981F, Spike N679K, Spike N764K, Spike N856K, Spike N969K, Spike P681H, Spike Q954H, Spike T95I, Spike T547K, Spike V143del, Spike Y144del, Spike Y145del, E T9I, M A63T, M D3G, N G204R, N P13L, N R203K, NSP2 I251M, NSP3 A1892T, NSP3 K38R, NSP3 L1266I, NSP3 S1265del, NSP4 T492I, NSP5 P132H, NSP6 I189V, NSP12 P323L, NSP14 I42V	227.78x
15	19.99	22.32	20.61	2022-01-17	South America / Brazil / Minas Gerais / Belo Horizonte	Female	73	EPI_ISL_10706106	VOC Omicron GRA (B.1.1.529+BA.)	BA.1.14	Spike D614G, Spike D796Y, Spike G142D, Spike G339D, Spike H655Y, Spike L981F, Spike N679K, Spike N764K, Spike N856K, Spike N969K, Spike P681H, Spike Q954H, Spike T95I, Spike T547K, Spike V143del, Spike Y144del, Spike Y145del, E T9I, M A63T, M D3G, M Q19E, N G204R, N P13L, N R203K, NSP2 I251M, NSP3 A1892T, NSP3 K38R, NSP3 L1266I, NSP3 S1265del, NSP4 T492I, NSP5 P132H, NSP6 I189V, NSP12 P323L, NSP14 I42V	309.39x
16	22.68	25.95	23.70	2022-01-17	South America / Brazil / Minas Gerais / Belo Horizonte	Male	71	EPI_ISL_10706107	VOC Omicron GRA (B.1.1.529+BA.)	BA.1.14	Spike D614G, Spike D796Y, Spike G142D, Spike G339D, Spike H655Y, Spike L981F, Spike N679K, Spike N764K, Spike N856K, Spike N969K, Spike P681H, Spike Q954H, Spike S371L, Spike S373P, Spike S375F, Spike T95I, Spike T547K, Spike V143del, Spike Y144del, Spike Y145del, E T9I, M A63T, M D3G, M Q19E, N G204R, N P13L, N R203K, NSP2 I251M, NSP3 A1892T, NSP3 K38R, NSP3 L1266I, NSP3 S1265del, NSP4 T492I, NSP5 P132H, NSP6 I189V, NSP12 P323L, NSP14 I42V	351.04x

CC BY

Sample	Ct E gene	Ct E RdRp	Ct N gene	Collection date	Collection location	Gender	Patient age	Accession ID GISAID	Lineage	Sublineage	AA Substitutions	Coverage
17	27.11	29.96	27.4	2022-02-18	South America / Brazil / Minas Gerais / Belo Horizonte	Male	63	EPI_ISL_11133066	VOC Omicron GRA (B.1.1.529+BA.)	BA.2.10	Spike D405N, Spike D614G, Spike D796Y, Spike G142D, Spike G339D, Spike H655Y, Spike K417N, Spike L84I, Spike N679K, Spike N764K, Spike N969K, Spike P681H, Spike Q954H, Spike R408S, Spike S371F, Spike S373P, Spike S375F, Spike T19I, Spike T376A, Spike V213G, E T9I, M A63T, M Q19E, N G204R, N P13L, N R203K, N S413R, NS3 T223I, NSP1 S135R, NSP3 G489S, NSP3 T24I, NSP4 L264F, NSP4 L438F, NSP4 T327I, NSP4 T492I, NSP5 P132H, NSP6 F108del, NSP6 G107del, NSP6 S106del, NSP12 P323L, NSP13 R392C, NSP14 I42V, NSP15 T112I	496.288x
18	21.35	24.62	21.95	2022-02-21	South America / Brazil / Minas Gerais / Belo Horizonte	Female	40	EPI_ISL_11133075	VOC Omicron GRA (B.1.1.529+BA.)	BA.2.9	Spike D405N, Spike D614G, Spike D796Y, Spike E484A, Spike G142D, Spike G339D, Spike H655Y, Spike K417N, Spike N501Y, Spike N679K, Spike N764K, Spike N969K, Spike P681H, Spike Q493R, Spike Q498R, Spike Q954H, Spike R408S, Spike S371F, Spike S373P, Spike S375F, Spike S477N, Spike T19I, Spike T376A, Spike T478K, Spike V213G, Spike Y505H, E T9I, M A63T, M Q19E, N G204R, N P13L, N R203K, N S413R, NS3 T223I, NSP1 S135R, NSP3 G489S, NSP3 T24I, NSP4 L264F, NSP4 L438F, NSP4 T327I, NSP4 T492I, NSP5 P132H, NSP6 F108del, NSP6 G107del, NSP6 S106del, NSP12 P323L, NSP13 R392C, NSP14 I42V, NSP15 T112I	746.904x
19	24.5	28.96	25.35	2022-02-23	South America / Brazil / Minas Gerais / Belo Horizonte	Female	79	EPI_ISL_11133082	VOC Omicron GRA (B.1.1.529+BA.)	BA.2.9	Spike D405N, Spike D614G, Spike D796Y, Spike E484A, Spike G142D, Spike G339D, Spike H655Y, Spike K417N, Spike N501Y, Spike N679K, Spike N764K, Spike N969K, Spike P681H, Spike Q493R, Spike Q498R, Spike Q954H, Spike R408S, Spike S371F, Spike S373P, Spike S375F, Spike S477N, Spike T19I, Spike T376A, Spike T478K, Spike V213G, Spike V622I, Spike Y505H, E T9I, M A63T, M Q19E, N G204R, N P13L, N R203K, N S413R, NS3 T12I, NS3 T223I, NSP1 S135R, NSP3 G489S, NSP3 T24I, NSP4 L264F, NSP4 L438F, NSP4 T327I, NSP4 T492I, NSP5 P132H, NSP6 F108del, NSP6 G107del, NSP6 S106del, NSP12 P323L, NSP13 R392C, NSP14 I42V, NSP15 T112I	691.909x
20	23.81	26.79	24.62	2022-02-25	South America / Brazil / Minas Gerais / Belo Horizonte	Male	41	EPI_ISL_11133089	VOC Omicron GRA (B.1.1.529+BA.)	BA.2.10	Spike D405N, Spike D614G, Spike D796Y, Spike G142D, Spike G339D, Spike H655Y, Spike K417N, Spike N501Y, Spike N679K, Spike N764K, Spike N969K, Spike P681H, Spike Q954H, Spike R408S, Spike S371F, Spike S373P, Spike S375F, Spike T19I, Spike V213G, Spike Y505H, E T9I, M A63T, M Q19E, N G204R, N P13L, N R203K, N S413R, NS3 T223I, NSP1 S135R, NSP3 G489S, NSP3 T24I, NSP4 L264F, NSP4 L438F, NSP4 T327I, NSP4 T492I, NSP5 P132H, NSP6 F108del, NSP6 G107del, NSP6 S106del, NSP12 P323L, NSP13 R392C, NSP14 I42V, NSP15 T112I	541.729x

The table shows the data of the samples used in this study. The samples were sequenced by Next-Generation Sequencing (NGS) in Illumina MiSeq platform. All samples were deposited in GISAID database. Pangolin (Pango v.4.0.4 PLEARN-v1.3) was used for de identification of SARS-CoV-2 variants.
